# Epigenetic repression of bone morphogenetic protein receptor II expression in scleroderma

**DOI:** 10.1111/jcmm.12105

**Published:** 2013-07-16

**Authors:** Yongqing Wang, Bashar Kahaleh

**Affiliations:** a Division of Rheumatology and Immunology, University of ToledoToledo, OH, USA

**Keywords:** epigenetics, BMPRII, scleroderma, vasculopathy, endothelial cells

## Abstract

Germline mutations in the bone morphogenetic protein type II receptor (BMPRII) gene play an essential role in the pathogenesis of familial pulmonary arterial hypertension (FPAH). In view of the histological similarities between scleroderma (SSc) and FPAH arterial lesion, we examined the expression levels of BMPRII in SSc microvascular endothelial cells (MVEC). Oxidative stress and serum starvation were used to examine apoptotic responses of MVECs. BMPRII expression levels were determined by RT-PCR and by Western blot. Epigenetic regulation of BMPRII expression was examined by the addition of epigenetic inhibitors to MVECs cultures, by methylation-specific PCR, and by sequence analysis of DNA methylation pattern of the BMPRII promotor region. SSc-MVECs were more sensitive to apoptotic signals than were normal-MVECs. A significant decrease in BMPRII expression levels in SSc-MVECs was noted, whereas no significant differences in the expression levels of BMPRIA and BMPRIB were observed. Similar reduction in expression levels was noted in SSc skin biopsies. The expression level of BMPRII in SSc-MVECs was normalized by the addition of 2-deoxy-5-azacytidine and trichostatin A to cell cultures. Extensive CpG sites methylation in the BMPRII promoter region was noted in SSc-MVECs with no detectable site methylation in control-MVECs. SSc-MVECs are more sensitive to apoptotic triggers than are control-MVECs. The enhanced apoptosis may be related to epigenetic repression of BMPRII expression as apoptosis of control-MVECs can be augmented by knocking down BMPRII expression. The role of BMPRII underexpression in the pathogenesis of SSc vasculopathy is suggested and should be investigated further.

## Introduction

Scleroderma (systemic sclerosis, SSc) is an autoimmune disease characterized by vascular injury and dysfunction, by excessive collagen deposition and by diffuse organ dysfunction. The pathogenesis of SSc has yet to be fully elucidated; nonetheless, the disease is to a large degree identified by the vascular features, particularly in its early stages. Disorganized microvasculature, endothelial apoptosis and vascular dysfunction occur early and evolve into a distinctive vasculopathy that is seen in all affected organs [1, 2]. The resulting arteriolar lesions are believed to be the basis for the major clinical complications in SSc, including digital ulcers, myocardial dysfunction, renal crisis and PAH [3].

Bone morphogenetic proteins (BMPs) are members of the TGF-β superfamily of proteins that coordinate cell proliferation, differentiation and survival. They exert their biological effects by interacting with serine/threonine kinase receptors BMP receptor IA (BMPRIA; ALK3) or BMPRIB (ALK6) and their coreceptor, BMPRII [4].

Recently, germline mutations in the bone morphogenetic protein type II receptor (BMPRII) were shown to be associated with familial and idiopathic PAH that manifests an arteriolar lesions similar to SSc vascular lesions [5, 6]. Recent investigations suggest that abnormal BMPRII signalling plays an important role in vascular cell proliferation and apoptosis and in the development of vasculopathy [7]. Reduced expression of BMPRII in many cases of sporadic PAH with no detectable BMPR mutations, including in SSc, has been described, suggesting that disease pathogenesis may involve an acquired underexpression of this gene [8, 9]. We therefore examined in this study the role of BMPRII in the pathogenesis of SSc vasculopathy.

## Methods

### Dermal microvascular endothelial cell isolation and cultures

After acquiring informed consent in compliance with the Institutional Review Board of Human Studies, a 5-mm skin biopsy was obtained from the affected skin (dorsal forearm) of six patients with diffuse cutaneous SSc of less than 3 years duration. All patients fulfilled the American College of Rheumatology criteria for the diagnosis of SSc, they were not on immunosuppressive or steroid therapy and none had digital ulcers or PAH. Microvascular endothelial cells (MVECs) were isolated from the biopsy samples and purified by CD31 magnetic beads as previously described [10] and cultured in Clonetics Endothelial Cell Basal Medium-2 (EBM-2) supplemented with EGM-2-MV growth factors (EGM-2) at 37°C in 5% CO_2_. Normal control dermal MVECs were similarly derived from healthy adult donors who were matched with the SSc patients for age, sex and race. The purity of isolated cells was >98% as determined by flow cytometry analysis using PE anti-human CD31 (BD-Pharmingen, Franklin lakes, NJ, USA). For serum starvation experiments, cells were cultured in DMEM medium without serum and growth factors for 24 hrs. All studies were performed on cells at passages 4–6.

### RNA extraction

Total RNA was extracted from either cultured cells or skin biopsy samples using the RNeasy Mini Kit according to the manufacturer's instructions (Qiagen, Valencia, CA, USA) in the presence of DNaseI. Skin biopsy samples were placed into 600 μl of buffer RLT (Qiagen RNeasy Mini Kit) and homogenized using a Pyrex tissue grinder. Total RNA was isolated using TRIzol Reagent according to the manufacturer's instructions (Invitrogen, Carlsbad, CA, USA). Contaminating genomic DNA was removed from the isolated RNA by treatment with amplification-grade DNase I (Invitrogen) for 2 hrs at 37°C. RNA was precipitated with 3 M NaAc (pH5.5) and 2.5 volumes of ethanol and quantified spectrophotometrically [11].

### Real-time quantitative PCR

RT-PCR was performed as described previously [12]. Briefly, first-strand cDNA synthesis was carried out using an RT-First Strand Kit (SABiosciences, Valencia, CA, USA) according to the manufacturer's instructions. Quantitative PCR was performed with a 7500 RT-PCR System (Applied Biosystems, Grand Island, NY, USA) using Power SYBR Green PCR Master Mix (Applied Biosystems). The differences in mRNA expression between samples were determined using the relative quantification method. The PCR program was conducted at 95°C for 10 min. as an initial polymerase activation step, followed by 40 amplification cycles at 95°C for 15 sec. and 60°C for 1 min. The cycle threshold (Ct) values of the samples were normalized to the Ct values of the housekeeping gene glyceraldehyde 3-phosphate dehydrogenase (GAPDH). The fold difference in gene expression of the samples was calculated using the equation 2−ΔΔCt. A list of the PCR primers used for each gene follows: GAPDH forward, 5′-TGCCA AA TATGATGACATCAAGAA-3′, GAPDH reverse, 5′-GGAGTGGGTGTC GCTG TTG-3′; BMPRII forward, 5′-TGGCAGT G AGGTCACTCAAG-3′, BMPRII reverse, 5′-TTGCGTT CATTCTGCATAGC-3′; BMPRIA forward, 5′-TTTATGGCACCCAAGGAAAG-3′, BMPRIA reverse, 5′-TGGTATTCAAGGG CACATCA-3′; BMPRIB forward, 5′-AAAGGTC GCTATG GGGAAGT-3′, BMPRIB reverse, 5′-GCAGCAATGAAACCCAAAAT-3′; Bcl-xl forward, 5′-ACCCCAGGGACAGCATATCA -3′, Bcl-xl reverse, 5′-TGGGATCCGACTCA CCAATA-3.

### Western blot analysis

Cell lysates were prepared as described previously [13]. Total protein concentration was determined by Bradford reagent as described by the manufacturer (Bio-Rad, Hercules, CA, USA). One hundred microgram of total proteins per sample was separated on 10% SDS-PAGE gels and transferred to polyvinylidene fluoride membranes. Protein detection was carried out with primary antibodies using goat anti-human BMPRII antibody (1:200; R&D systems, Minneapolis, MN, USA) or monoclonal antibodies against β-actin (Abcam Inc., Cambridge, MA, USA) or β-tubulin (Santa Cruz Biotechnology, Dallas, TX, USA). Bands were visualized with the corresponding horseradish peroxidase-conjugated secondary antibodies (Santa Cruz Biotechnology) and detected using the enhanced chemiluminescence (Pierce, Rockford, IL, USA), followed by autoradiography. Relative quantification was performed by normalization with β-actin or tubulin.

### Cell viability assay (MTT assay)

Cell viability was quantified by the MTT assay using the CellTiter Nonradioactive Cell Assay Kit (Promega, Madison, WI, USA) according to the manufacturer's instructions. The values were expressed as percentages over those of control [14].

### Annexin V staining and flow cytometry analysis of cell apoptosis

Cells were incubated with Annexin-V-Fluorescein (green fluorescence) in a Hepes buffer containing PI (Propidium, red fluorescence) and examined under fluorescence microscopy using the Annexin-V-Fluos Staining Kit (Roche, Indianapolis, IN, USA ). The Annexin V-FITC Apoptosis Detection Kit I (BD Biosciences, San Jose, CA, USA) was used to detect apoptosis by flow cytometry (Guava PCA). Cells were harvested and processed according to the manufacturer's instructions.

### Knockdown of BMPRII by siRNA

Human BMPRII-specific validated Stealth siRNA oligonucleotides, the Stealth-negative control LG GC (IR-siRNA) and siRNA fluorescence-positive control were all obtained from Invitrogen and transfected into MVECs using the Basic Nucleofector Kit for Primary Mammalian Endothelial Cells (Amaxa, Cologne, Germany) according to the manufacturer's instructions. After 24 hrs, the cells were collected for Flowcytometry and real-time PCR assays to examine the transfection efficiency and knockdown effects [15].

### Methylation-specific PCR and bisulphited DNA sequencing

Genomic DNA was extracted from SSc and control-MVECs using the DNAeasy Qiamp Mini Kit (Qiagen) according to the manufacturer's instructions. DNA modification was performed with the CpG Modification Kit (Qiagen) according to the manufacturer's instructions. Two microgram of genomic DNA from each sample was modified by sodium bisulphite treatment, which converts all unmethylated cytosines to uracils and leaves the methylated cytosines unchanged. Modified DNA was amplified according to the method described [16]. The primers for the BMPRII Methylation-specific PCR (MSP) were as follows: unmethylated primers 5′-GGTGGAATTTATTTTAGGTAAGATTGA-3′ (forward), 5′-AAAAAATTAAAAAAACCC TAACAAA-3′ (reverse); methylated primers 5′-TGGAATT TATTTTAGGTAAGATCGA-3′ (forward), 5′-AAAAAATTAAAAAAACCCTAACGAA-3′ (reverse); wild-type primers 5′-GTGGAATTTACCTCAGGCAAGATCG-3′ (forward), 5′-GAGGGTTGGGGAGGCCCTG GCGAAG-3′ (reverse). The primers for the sequencing of bisulphite BMPRII promoter were 5′-GTTAGGGTTTTTTTAATTTTTTTA-3′ (forward), 5′-AACCTAACTACAAAACTT TCATACC-3′ (reverse). The amplified fragments were inserted into pCR2.1 vector. Sequencing was performed with the ABI DNA sequencer, Model 310 (Perkin-Elmer, Waltham, MA, USA ).

### Images intensity quantification

The software Scion Image, beta 4.03, was downloaded from http://www.Scioncorp.com and used to quantify the specific bands in Western blots.

### Statistical analysis

Mean and SD values were calculated, and the Mann–Whitney *U*-test was used for analysis of statistical significance. *P* values less than 0.05 were considered significant.

## Results

### BMPRs expression level in control and SSc endothelial cells and skin biopsies

**Fig. 1:**
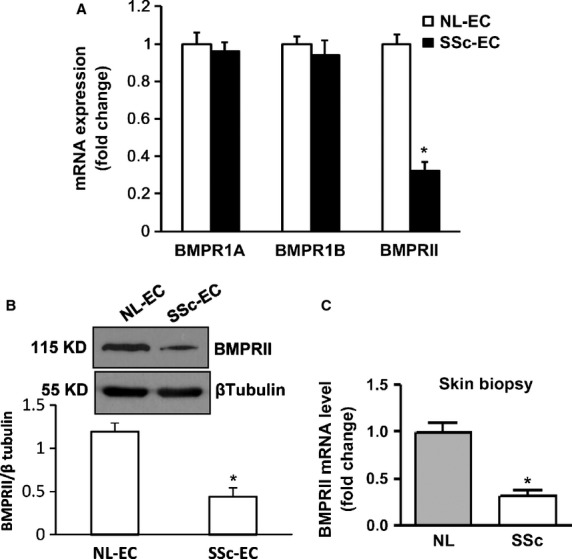
Decreased expression levels of BMPRII in systemic sclerosis (SSc) cells and skin. (**A**) mRNA expression levels of all BMPR family members (BMPR1A, BMPR1B and BMPRII) in normal-MVECs and SSc-MVECs were measured by SYBR Green real-time PCR analysis. Values are the fold increase/decrease compared with expression in the normal control group (ascribed an arbitrary value of 1). There was a significant decrease in BMPRII expression levels in SSc-MVECs *P < 0.01 *versus* normal control (*n* = 5 cell lines obtained from five different cases). (**B**) The BMPRII protein expression level was measured by Western blot analysis. The expression levels were quantified using tubulin levels for normalization. The level of BMPRII was significantly decreased in SSc-MVECs *P < 0.01 *versus* normal control (*n* = 3 different cell lines, (**B**) it is a representative finding from one cell line). (**C**) BMPRII expression levels in normal and SSc skin biopsy samples were measured by SYBR real-time PCR. Expression of BMPRII in fresh skin biopsy samples from SSc patients (*n* = 3) was down-regulated as compared with healthy normal control samples (*n* = 3, *P < 0.01). Values are the mean ± SD.

The expression levels of BMPR1A, BMPR1B and BMPRII were measured in both control-MVECs and SSc-MVECs skin biopsies by quantitative reverse-transcription PCR (qPCR) and by Western blot analysis. BMPRII expression was significantly down-regulated in SSc-MVECs both at the mRNA level (Fig. 1A; 0.35 ± 0.13, *P* < 0.01 *versus* control) and at the protein level (Fig. 1B). There was no difference in the expression levels of BMPR1A and BMPR1B between SSc-MVECs and control-MVECs (Fig. 1A). BMPRII expression levels were also measured in freshly isolated RNA obtained from 5-mm skin biopsies (*n* = 3 SSc and three control cases). Significant reduction in BMPRII expression levels was noted in SSc skin samples (Fig. 1C; 0.28 ± 0.12, *P* < 0.01 *versus* control samples), confirming that BMPRII expression levels are reduced in SSc tissue.

### MVECs response to apoptotic signals

**Fig. 2:**
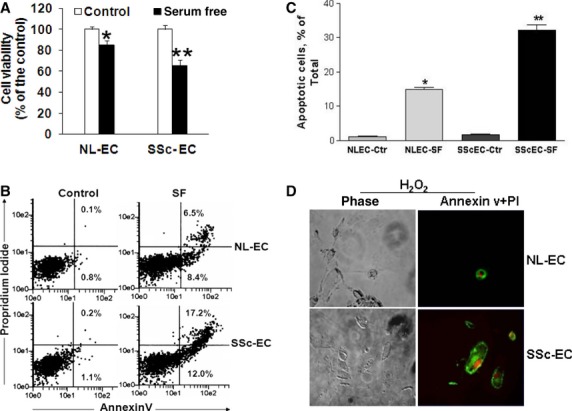
MVECs apoptosis. (**A**) Normal-MVECs and SSc-MVECs were subjected to serum and growth factors withdrawal (SGF) for 24 hrs. Cell viability was measured by CellTiter Proliferation Assay. Viability of untreated control cells was set at 100%. Data are expressed as mean ± SD from five experiments. *P < 0.05 *versus* NL-EC ctr; **P < 0.05 *versus* SSc-EC ctr and NL-EC ctr. (**B**) Guava PCA analysis of apoptotic MVECs. Normal-MVECs and SSc-MVECs were labelled with Annexin V-FITC and propidium iodide, and apoptosis was assessed by Guava PCA. Data from five experiments showed that SSc-MVECs were more sensitive to SGF-induced apoptosis. *P < 0.05 *versus* normal control; **P < 0.05 *versus* SSc-MVECs control and SGF-treated normal-MVECs. (**C**) Normal and SSc-MVECs were treated with H_2_O_2_ 400 μM for 24 hrs. (**D**) Cells were stained with Annexin V and PI (Propidium iodide) using Annexin V Fluos Staining Kit.

Control-MVECs and SSc-MVECs were subjected to serum and growth factor withdrawal (SGFW) or to H_2_O_2_ 400 μM for 24 hrs to test the differential response to apoptotic signals. Apoptosis was determined by Annexin V staining and by cell viability assay. SGFW resulted in significantly lower SSc-MVECs cell viability than control-MVEC cell viability (85.12 ± 4.24% in control-MVECs and 67.24 ± 5.28% in SSc-MVECs, mean ± SD of quadruplicate experiments, Fig. 2A, *P* < 0.05). Similarly, increased SSc-MVEC susceptibility to apoptosis was seen using Annexin V staining measured by flow cytometry. SGFW treatment significantly increased Annexin V staining of cells from 1.21 ± 0.31% to 14.87 ± 1.3% in control-MVECs and from 1.80 ± 0.36% to 32.19 ± 2.84% in SSc-MVECs (Fig. 2B, *P* < 0.05). Similar results were obtained after the addition of H_2_O_2_ for 24 hrs (Fig. 2C and D), confirming that SSc-MVECs are more vulnerable to apoptotic stimuli than control-MVECs.

### BMPRII signalling effects on MVECs apoptosis

**Fig. 3:**
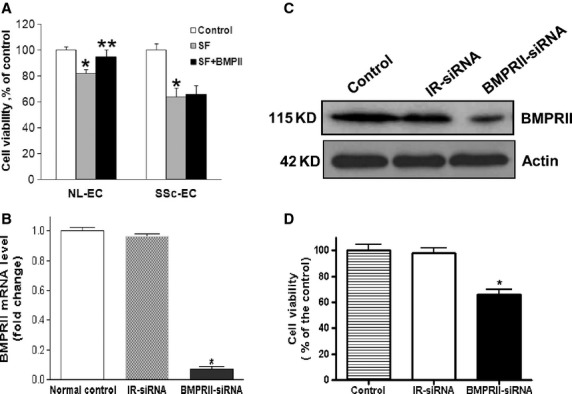
Effects of BMP2 and reduced BMPRII expression on MVECs survival. (**A**) BMP2 shows the anti-apoptotic effects on starvation-induced apoptosis in normal-MVECs, but not in SSc-MVECs. Normal and SSc-MVECs cells were cultured in serum and growth factor free (SGF) medium with and without BMP2 (200 ng/ml) for 24 hrs. The cell viability was measured by CellTiter Proliferation Assay. Viability of untreated control cells was set at 100%. Data are expressed as mean ± SD from triplicate experiments, *P < 0.01 *versus* control; **P < 0.05 *versus* SGF without BMP2 treatment. (**B**–**D**) Knockdown of BMPRII in normal-MVECs by siRNA led to sensitize the cells to apoptotic stimuli. (**B** and **C**) reduced BMPRII mRNA and protein expression by siRNA. Normal-MVECs were transfected with IR-iRNA or BMPRII-siRNA. After 24 hrs, BMPRII mRNA expression levels were measured by real-time PCR (see **B**; the data are normalized for GAPDH, data are presented as mean ± SD, *n* = 5, *P < 0.01 *versus* control and IR-siRNA control), and BMPRII protein expression levels were assessed by Western blot analysis (**C**). (**D**) Effect of reduced BMPRII expression on MVECs survival. Normal endothelial cells were transfected with IR-siRNA or BMPRII-siRNA and cultured in EBM-2 medium without FCS and growth factors for 24 hrs (SGF). Cell viability was determined using the CellTiter 96 Cell Proliferation Assay. Data showed that reduction in BMPRII expression in Normal-MVECs significantly increased cell apoptosis induced by SGF. *P* < 0.01 *versus* control and IR-siRNA control.

The addition of BMP2 at 200 ng/ml to cells subjected to SGFW resulted in a significant increase in control-MVECs viability (from 82 ± 5.2% to 94.8 ± 5.4%, mean ± SD, *P* < 0.05, Fig. 3A), but had insignificant effect on SSc-MVECs viability. To investigate whether the protective effects of BMP2 on control cells were indeed mediated through BMPRII signalling, we used specific silencing siRNA to knock down BMPRII expression in normal-MVECs. After 24 hrs of transfection with siRNA, >90% knockdown of BMPRII mRNA was observed by RT-PCR (Fig. 3B), and >50% reduction in BMPRII protein expression (Fig. 3C) was observed by Western blot analysis. However, there was no effect for the irrelevant siRNA on the expression levels (Fig. 3B and C). Reduction in BMPRII expression levels using specific siRNA in control-MVECs significantly decreased the level of cell viability induced by SGFW (56 ± 4% mean ± SD, in BMPRII knocked down normal-MVECs *versus* 80 ± 6% in control-MVECs, *P* < 0.05, Fig. 3D).

### BMPRII signalling upregulates the expression level of the anti-apoptotic gene Bcl-xL

**Fig. 4:**
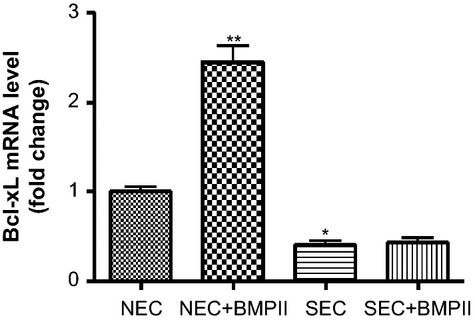
The effects of BMP2 on anti-apoptotic gene Bcl-xL mRNA. Normal and SSc-MVECs cells were treated with BMP2 (200 ng/ml) for 24 hrs. The mRNA expression levels of Bcl-xL were measured by SYBR Green real-time PCR analysis. The expression level of Bcl-xL was markedly increased in BMP2-treated normal-MVECs, but not in SSc-MVECs. Values are the fold changes compared with expression in the normal control group (ascribed an arbitrary value of 1) and are expressed as mean ± SD from triplicate experiments.*P < 0.05, **P < 0.01 *versus* normal control.

To explore how the BMPRII signalling pathway protects the cells from apoptosis, normal-MVECs and SSc-MVECs were treated with BMP2 (200 ng/ml) for 24 hrs, and Bcl-xL mRNA expression was measured by qPCR. The addition of BMP2 to control-MVECs led to a 2.42 ± 0.16-fold increased expression of Bcl-xL, whereas in SSc-MVECs, the expression level of Bcl-xL was reduced significantly at baseline (mean ± SD, 0.47 ± 0.08, *P* < 0.01, compared with the normal control, and failed to increase after the addition of BMP2 at 200 ng/ml (Fig. 4), 300 or even 400 ng/ml (data not shown).

### BMPRII promoter CpG island methylation in Scleroderma endothelial cells

**Fig. 5:**
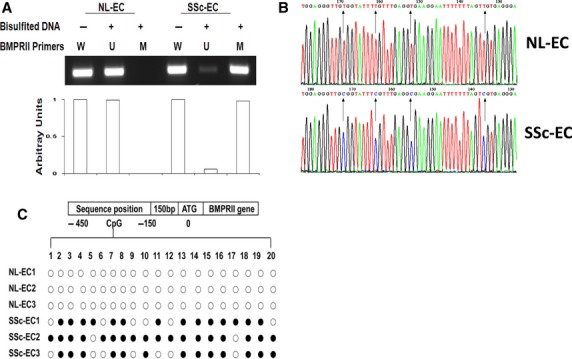
Methylation of the promoter region of BMPRII in SSc-MVECs. (**A**) PCR and methylation-specific PCR (MSP) were performed with genomic DNA from normal and SSc-MVECs. Lanes 1 and 4, PCR using unmodified DNA and wild-type primers (W); lanes 2 and 5, MSP using bisulphited DNA and unmethylated primers (U); lanes 3 and 6, MSP using bisulphited DNA and methylated primers (M). The separated DNA fragments are shown (upper panel), and results were quantified using the normal PCR products as control (ascribed an arbitrary value of 1) (lower panel, mean of triplicate experiments). (**B**) Representative sequences of the promoter region of the BMPRII gene after bisulphite treatment. DNA was isolated from normal and scleroderma MVECs. After sodium bisulphite treatment, the DNA was subjected to PCR and sequenced. The figure depicts the sequence of part of the promoter region of the BMPRII gene. The arrows indicate CpG sites. Adenine (A) peaks are green, cytosine (C) peaks are blue, guanine (G) peaks are black and thymine (T) peaks are red. (**C**) The methylation status of the CpG sites in the BMPRII promoter region (−150 to −450). The cytosines in the CpG sequences −175, −206, −208, −226, −231, −235, −243, −245, −257, −319, −329, −376, −383, −391, −396, −402, −409, −429, −436 and −445 are coded from 1 to 20 respectively. Open circles indicate no methylation; solid circles indicate CpG island methylation. Six genomic samples (three from normal endothelial cells and three from SSc endothelial cells) were sequenced.

The proportion of methylated and unmethylated CpG islands in the promoter region of the BMPRII gene was examined by MSP after DNA bisulphite modification. The primers for MSP were designed using MethPrimer (http://www.urogene.org/methprimer) to amplify a sequence from −842 to −648). Methylation of the BMPRII promoter region was noted in SSc-MVECs (mean ± SD of methylated to wild-type amplicons: 0.98 ± 0.026), with no detectable methylation products in control-MVECs (Fig. 5A). To confirm hypermethylation of the BMPRII promoter region, we cloned and sequenced the BMPRII promoter region −150 to −450 of bisulphited genomic DNA. Dense methylation in the predicted location of CpG islands in the SSc-MVEC promoter region was noted in DNA derived from three SSc cell lines, whereas no methylation was noted in three matched control cell lines (Fig. 5B and C). There are 20 CpG sites in the sequenced BMPRII fragment; among them, five sites overlap with transcription factors–binding sites as revealed by transcription search ‘TFSEARCH’ analysis. Two of these CpG sites are potentially important in the regulation of BMPRII transcription. One is a CG-containing SP1-binding site, and the other is a CG-containing EGR-binding site. Both sites are methylated in all three SSc samples and they correspond to the 4th, 13th CGs (Fig. 5C). It has been reported that methylation of these sites can hinder transcriptional factor binding and that transcription factor binding to these sites is essential for the transcriptional regulation of BMPRII [17, 18].

### Effects of epigenetic inhibitors on BMPRII expression

**Fig. 6:**
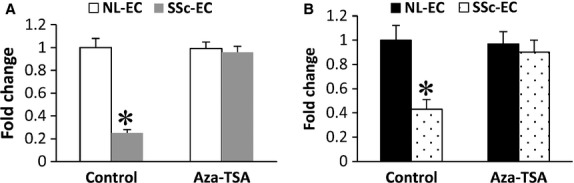
Modulation of BMPRII expression by epigenetic DNA modifications. Normal and SSc-MVECs were treated with 5 μM of 2-deoxy-5-azacytidine (5-Aza) for 5 days and 100 ng/ml of trichostatin (TSA). The BMPRII mRNA expression was measured using real-time polymerase chain reaction. Values are the fold increase/decrease compared with expression in the normal control group (ascribed an arbitrary value of 1) and are expressed as mean ± SD from triplicate experiments. *P < 0.01 *versus* normal control, Aza-TSA treated SSc-MVECs (**A**). BMPRII protein expression level was measured by Western blot analysis. The expression levels were quantified using tubulin levels for normalization. The level of BMPRII was significantly decreased in SSc-MVECs *P < 0.01 *versus* normal control (*n* = 3 different cell lines (**B**).

To investigate whether an epigenetic mechanism mediates underexpression of BMPRII in SSc-MVECs, control-MVECs and SSc-MVECs were treated with the DNA methyltransferase inhibitor (5-Aza-2′-deoxycytidine) at 5 μM for 5 days and the histone deacetylase inhibiter (TSA, 100 ng/ml of trichostatin) alone or in combination. Addition of either inhibitors alone resulted in modest increase in BMPRII expression (data not shown); however, the expression levels of BMPRII in SSc-MVECs increased and reached normal levels when both were added to cell cultures (levels increased from 0.26 ± 0.03 to 0.96 ± 0.05; *P* < 0.01, SSc-MVECs untreated control *versus* Aza-TSA-treated SSc-MVECs), while no effects on BMPRII expression levels were noted in control-MVECs (Fig. 6). Moreover, SSc-MVEC apoptotic response to SGFW was identical to the control-MVEC response after the addition of Aza-TAS (cell viability 83 ± 4.2% in control-MVECs *versus* 81 ± 6.7% in SSc-MVECs, mean ± SD, data not shown).

## Discussion

Scleroderma (SSc) is a systemic autoimmune disease of unknown aetiology and pathogenesis. It is characterized by progressive vasculopathy and widespread tissue fibrosis. The disease is, to a large degree, defined by vascular features, particularly in its early stages. Vascular injury is an early event in scleroderma that precedes tissue fibrosis and involves the small vessels, particularly the arterioles [19, 20]. Swelling of the intima and intimal proliferation with mononuclear cell infiltration is seen in the small arterioles that evolve into a distinctive fibroproliferative arteriolar disorder with intimal and medial thickening and adventitial fibrosis [21]. The intimal proliferation is of a uniform and symmetrical nature that forms a neointima indistinguishable from that formed in many other disorders, including familial pulmonary arterial hypertension lesion.

Endothelial cell apoptosis was first described on ultrastructural examination of SSc biopsies in the early stages of the disease in association with the inflammatory stage, suggesting a causal association [20]. It was later noted in the University of California at Davis lines 200/206 chickens that spontaneously develop an SSc-like disease [2]. There is evidence to suggest that infectious agents, autoantibodies, sheer stress, ischaemia/reperfusion or interferons potentially initiate and/or perpetuate the cascade of vascular damage and remodelling.

In this study, we examined the expression level of BMPRII and MVEC apoptosis in SSc in view of the recent description of the crucial role of BMPRII signalling in the development of the arterial lesion in PAH. BMPs are members of the TGF-β family, which regulates cell proliferation, apoptosis and differentiation [22]. Three BMP receptors are identified: IA, IB and II. The ligands for the BMPR represent a family of secreted growth factors known as the BMPs. Signal transduction through this pathway involves heterodimerization of BMPRII with BMPR1, which results in phosphorylation of BMPRI, initiating activation of signal transduction [23]. BMPs have pleiotropic effects depending on the cell type, the specific ligand and the environmental milieu. For example, BMPs can inhibit proliferation and induce apoptosis in human pulmonary artery smooth muscle cells (SMCs); however, in other cell types, including cardiomyocytes and endothelial cells, signalling *via* this pathway can have an opposite effect that promotes cell survival [24]. In this report, we show that the expression level of BMPRII is significantly reduced in SSc-MVECs and SSc skin and that SSc cells are more vulnerable to apoptosis induced by serum starvation and oxidation injury. Moreover, the addition of BMP2 decreased apoptosis in response to serum deprivation in normal-MVECs, but not in SSc-MVECs, whereas the knockdown of the BMPRII using siRNA increased the basal level of apoptosis in normal-MVECs to levels similar to those seen in SSc cells. The addition of BMP2 to normal EC cultures led to upregulation of the anti-apoptotic gene Bcl-xL, but not in SSc cells. It is known that BMPRII signalling lead to stabilization of XIAP through phosphorylation of Akt, which inhibits caspase -9 and -3, and through TAK1, which activates NF-kB, leading to increase expression of the anti-apoptotic gene Bcl-xL [25, 26].

Most investigations have focused on the potential importance of BMPRII mutations on SMCs as it is clear that BMP signalling represents an inhibitory pathway and prevents excessive pulmonary arterial muscularization by reducing SMCs growth and increasing apoptosis. Still, BMPRII is mainly expressed by the vascular endothelium, suggesting that BMPRII defect in the endothelium is a crucial event in the evolution of proliferative vascular disorder. This suggestion is supported by the fact that genetic ablation of BMPRII gene restricted to the pulmonary endothelium clearly leads to the development of PAH, which implicates the endothelium as a critical target in the molecular pathogenesis of the proliferative vasculopathy in PAH pathogenesis [27]. Our results strongly suggest that BMPs protect against apoptosis in MVECs, which supports the role of the BMPRII pathway signalling in endothelial survival. Therefore, the underexpression of BMPRII in SSc may lead to increased MVEC loss in response to environmental triggers encountered in the disease. Defective *BMPRII* signalling may contribute to disease pathogenesis by the reported enhanced expression and availability of the cytokines endotheline-1(ET-1) and transforming growth factor beta (TGF-β), both of which are implicated in disease pathogenesis and are unregulated in SSc and PAH. Thus, *BMPRII* knockdown in pulmonary microvascular endothelial cells results in significantly increased ET-1 levels in the cell supernatant. A role for TGF-β is suggested since that increase was prevented by *ALK2* knockdown. Thus, increased ET-1 production by endothelial cells as a consequence of *BMPR II* dysfunction may be clinically relevant in the pathogenesis of the vasculopathy in PAH and SSc [28, 29]. The loss of small precapillary arteries is a prominent feature of PAH and SSc vasculopathy that is thought to be as a result of enhanced susceptibility of ECs both to apoptosis and to failure of angiogenesis. The data suggest that BMPRII signalling supports endothelial cell viability by recruiting Wingless (Wnt) signalling pathways to promote endothelial proliferation, survival, and motility and by the recruitment of Disheveled (Dvl) to promote RhoA-Rac1 signalling necessary for EC motility and angiogenesis. These findings suggest that the recruitment of both canonical and non-canonical Wnt pathways is required in BMPRII-mediated angiogenesis [30, 31].

It is known that defective expression of BMPRII alone is not sufficient to cause vascular lesions, as shown by the reduced penetrance of BMPRII mutation for PAH to ∼10–20% of affected individuals. The reason is likely to be related to the requirement for yet another ‘hit’, as in other genetic factors or environmental triggers. This requirement was shown by the development of PAH in individuals with BMPRII mutation after exposure to the appetite suppressant amfepramone or after exposure to hypoxia [32, 33]. In this report, we tested the effect of oxidation injury on MVECs apoptosis. Addition of H_2_O_2_ resulted in significant MVECs apoptosis, particularly in SSc cells that were reversed by addition of BMP2 in normal cells, but not in SSc cells, suggesting a role for BMPRII signalling in preventing oxidation-induced apoptosis in normal cells and that a defective expression of BMPRII in SSc renders the cells susceptible to oxidation injury. Thus, it seems likely that oxidation injury may represent the additional ‘hit’ required for the development of vasculopathy on a background of reduced BMPRII expression. Although the pathogenesis of SSc remains unknown, oxidative stress and high levels of reactive oxygen species have been directly or indirectly implicated in disease pathogenesis [34, 35]. SSc patients exhibit significant evidence for oxidative stress, which is shown by abnormalities of nitric oxide and nitric oxide synthase and by increased levels of oxidative biomarkers [36, 37]. Moreover, high levels of reactive oxygen species production by fibroblasts, independent of inflammatory stimulus, suggest that this cell type is the endogenous source for oxidative stress in SSc [38]. In this study, we show that oxidation may trigger apoptosis in cells that underexpress BMPRII.

The current data suggest that an epigenetic mechanism is involved in repressing BMPRII expression in SSc-MVECs. In support of this conclusion is first the finding that the addition of inhibitors of DNA methyltransferases and histone deacetylases led to normalization of the expression levels. Second, MSP analysis showed the presence of methylated fragments in SSc samples only. Third, extensive CpG site methylation in the BMPRII promoter region is shown in SSc-MVECs, whereas no methylation was noted in control-MVECs. Epigenetic modifications of the genome provide a mechanism that allows the stable propagation of gene activity states from one generation of cells to the next. Because epigenetic states are reversible, they can be modified by environmental factors, which may contribute to the development of abnormal cellular phenotypes.

It was proposed in 1975 that DNA methylation might be responsible for the stable maintenance of a particular gene expression pattern through mitotic cell division. Since then, ample evidence has been obtained to support this assertion, and DNA methylation is now recognized as a chief contributor to the stability of pathological gene expression states [39]. Dysregulation of epigenetic control is characteristic of a growing number of human diseases, most prominently in cancer and in human vascular disorder characterized by endothelial dysfunction [40], as seen in SSc. It is increasingly appreciated that epigenetic pathways are responsive to a wide variety of internal and external environmental stimuli, including oxidative, immune and infectious stimuli [41].

The current findings may have significant impact on the understanding of the pathogenesis of SSc vasculopathy and may have significant therapeutic implications as neither methylation of a gene nor alteration in chromatin structure is irreversible. Because of this potential for reversibility, epigenetic gene regulation is theoretically amenable to intervention. Thus, a better understanding of the role of epigenetics in SSc may lead to the development of a novel therapy.
